# Health cadres empowerment program through smartphone application-based educational videos to promote child growth and development

**DOI:** 10.3389/fpubh.2022.887288

**Published:** 2022-10-13

**Authors:** Dewi Friska, Aria Kekalih, Fergie Runtu, Andini Rahmawati, Naufal Arkan Abiyyu Ibrahim, Eghar Anugrapaksi, Nindya Permata Bunda Surya Utami, Alya Darin Wijaya, Rathia Ayuningtyas

**Affiliations:** ^1^Department of Community Medicine, Faculty of Medicine, Cipto Mangunkusumo Hospital, Universitas Indonesia, Jakarta, Indonesia; ^2^Faculty of Medicine, Universitas Indonesia, Jakarta, Indonesia; ^3^Lebak Bulus Primary Health Center Urban Village, Jakarta, Indonesia

**Keywords:** health cadres, empowerment program, educational videos, smartphone application, child health

## Abstract

Indonesia's health care system relies on non-health professionals called cadres to operate child health promotion programs in the Public Health Center (Puskesmas). Despite this effort, the child malnutrition rate remains high. This study aimed to identify and develop health promotion media that can assist health cadres in promoting child health. This study was divided into three-phase. The first phase was the need assessment using focus group discussion and knowledge, attitude, and practice (KAP); the second phase is video development, which involves medical students, general practitioners, pediatricians, and health promotion experts; and the third phase was video viewing by cadres and post-viewing tests for health cadres. A comparison of pre-test and post-test participants' total scores was performed with the student's *T*-test. Need assessment showed that the knowledge of the cadres needs improvement and there was a need for proper educational media material that can be used by the Puskesmas. Five videos were produced, four videos were about children's nutritional intake recommendations during four different age groups and one video was about the information and invitation to come to Integrated Health Service Post (Posyandu). There was a significant improvement from pre-test total scores to post-test total scores (*p* < 0.001). Smartphone application-based educational videos are effective and reliable child health promotion media for Puskesmas staff and parents.

## Introduction

Indonesia's healthcare system relies not only on healthcare professionals but also on non-professionals called the health cadres. They are community volunteers who have been trained by healthcare professionals to operate and aid in health promotion programs run by the Public Health Center (Puskesmas). Posyandu, an integrated health service post dedicated to promoting maternal and child health, is one such program. In Posyandu, health cadres conduct nutritional screening for children and pregnant women once per month. Nutritional status was plotted on a growth chart in each child's personal maternal and child health book released by Indonesia Health Ministry and reported to the Puskesmas. Posyandu also provides health promotion through education, primarily on nutrition for attending parents using flipcharts or maternal child health books (1). Education and child monitoring in Posyandu by health cadres was one of the success key factors in reducing stunting in some districts in Indonesia, as reported by the World Bank and Ministry of Health. However, cadre capacity needs support to provide better nutrition education, in the form of training and educational tools. One of the important aspects of a health promotion program is empowerment. In this program, researchers develop training material to empower health cadres as community educators on the topic of child nutrition (2).

Child malnutrition remains a national burden in Indonesia for the past decade. National under-five stunting was reported to be around 37%, higher than the average stunting rate of developing countries at 25% (3). Despite government efforts to tackle stunting issues through health promotions, little progress was evident in lowering the national stunting rate. The first 1,000 days are the critical window period for health intervention (4). Nutrition during this period not only impacts the child's growth and development but also has medical and socioeconomic consequences in adulthood. Stunting, an outcome of growth failure in the first 1,000 days of life, is strongly associated with lower human capital. Adequate dietary intake and appropriate nutritional practice become essential in reducing stunting (4, 5). A recent review also found evidence suggesting maternal education, non-exclusive breastfeeding, and low socioeconomic status as significant stunting determinants (6). Based on these findings, those most vulnerable to stunting are children in low-socioeconomic status households, due to limited access to information and health care. These populations are also the ones that are more likely to rely on health cadres to access healthcare. Therefore, it is imperative to equip health cadres with the optimal tools and knowledge in reaching these vulnerable populations.

Telehealth by definition is the delivery of health care services, including patient education and health information, through a distance by utilization of information and communication technology. In 2018, around 40% of Indonesia's population was internet-users, accompanying this growth of internet-user is a burgeoning telehealth industry. Patient education using telehealth applications was an example of health cadre empowerment that has proven effective to deliver more informative nutrition messages to mothers and child-caring families (7–9). Indonesian Pediatric Association has caught up with the trend by developing an application called Primaku^®^. This application facilitates parents to understand their children's growth and development and provides health education in childcare. Primaku^®^ intends to expand their users to include health cadres, to help them in children's growth and development surveillance and health promotion. This study aimed to identify and develop health promotion media that can assist health cadres through an existing mobile application platform in educating parents on children's nutrition in the first 2 years of life as part of a community empowerment program. Specific objectives included determining the most suitable health promotion media through discussion and survey, creating a trustworthy media by reviewing literature and content, and testing the efficacy of the new health promotion media with pre-test and post-test.

## Methods

### Subjects and study design

**Figure 1:**
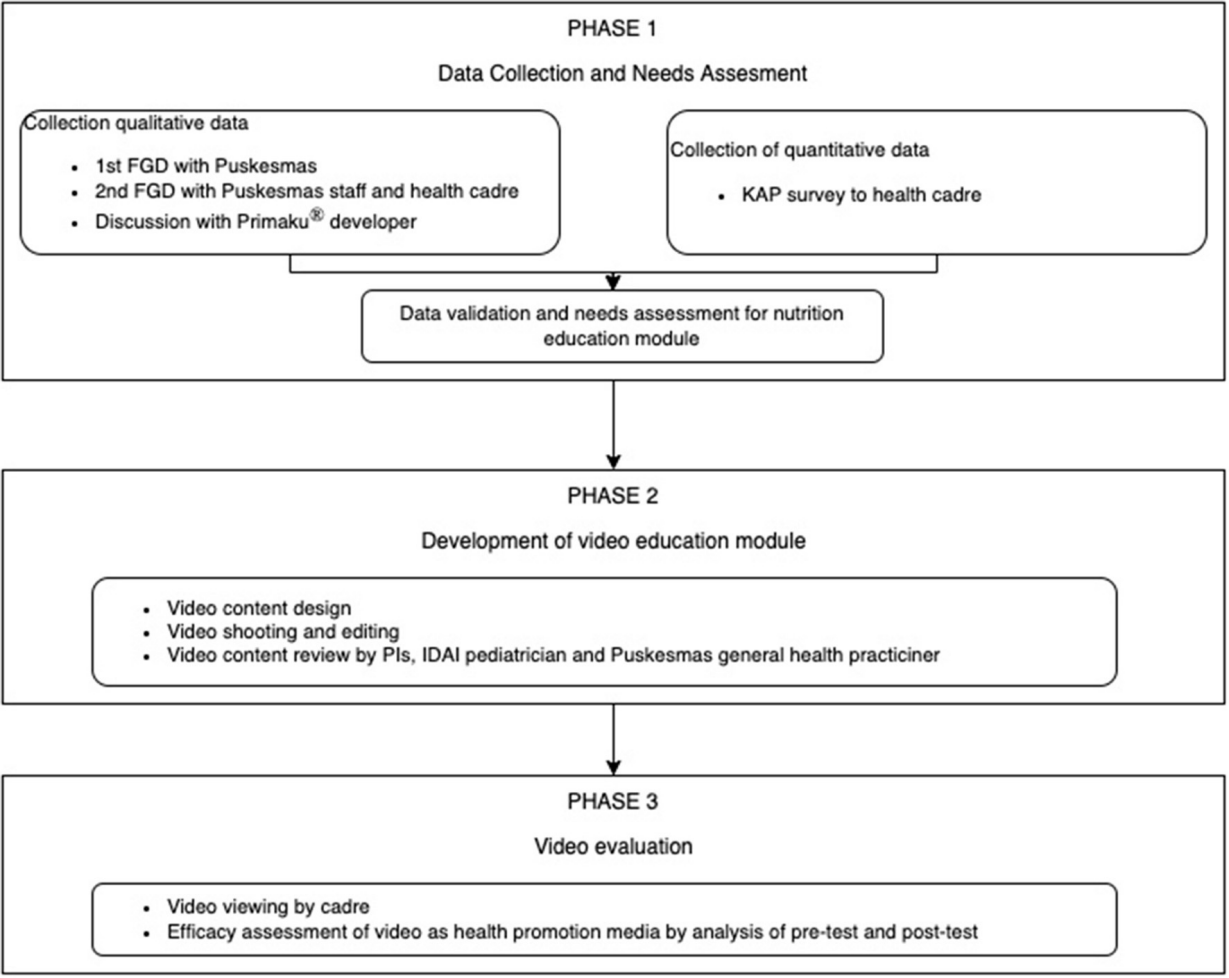
Three phases of the study: data collection and needs assessment, development of video education module, and video evaluation.

This study was a mixed-method study design consisting of qualitative data collection through focus group discussion (FGD) and quantitative analysis from baseline knowledge surveys, pre-test, and post-test. Researchers used concurrence triangulation methods to collect the data regarding the health cadre's need for nutrition education topic, perspective on their current nutrition knowledge, and how the mobile application might support their work in educating mothers and their families. This study was divided into three-phase as described in Figure 1. For the first phase, researchers recruited the participants from Puskesmas staff involved in the health promotion and nutrition program for the first FGD. Representatives from sub-district Puskesmass under Cilandak district with health cadres in that sub-districts were invited for the second FGD and baseline knowledge, attitude, and practice (KAP) survey. Data from phase 1 was used to develop educational media and topics in phase 2 activities. For the third phase, we reinvited health cadres from all sub-district areas in the Cilandak district. The team used the purposive sampling method to recruit participants. The study was led by two principal investigators (PI) from the Department of Community Medicine Universitas Indonesia in collaboration with one general practitioner (GP) from Lebak Bulus district Puskesmas, in coordination with Cilandak district Puskesmas. This study is a part of a health promotion program. After a socialization meeting and obtaining a permit from the Head of Cilandak Public Health Center, the program was launched.

The study was conducted in Puskesmas of Cilandak Sub-district, Jakarta, Indonesia. The Puskesmas is located in Lebak Bulus urban village in Cilandak sub-district, South Jakarta. Jakarta is the metropolitan capital city of Indonesia. The city is a bustling center of commerce, with residents of varying economic statuses. Lebak Bulus Urban Village encompasses an area of 411.42 Ha with 42,600 inhabitants. In total 17% of the population of Lebak Bulus 7,351 are women of productive age (20–39 years). In terms of livelihood, 24.94% of the population works as employees in the government and private sectors. The education level of the population also varies. Most residents (40%) graduated from senior high school. Only 13% of residents are university graduates.

The needs assessment phase involved 30 health cadres, 5 Puskesmas staff of the Cilandak Sub-district Puskesmas, and PrimaKu^®^ developer. The video development phase involves medical students, general practitioners, pediatricians, and health promotion experts. There were 20 health cadres participating in the video evaluation phase.

### Phase 1: Needs assessment

The initial assessment was carried out to determine the general overview of child's nutritional health status and evaluate health promotion programs in Cilandak sub-district Puskesmas. We qualitatively gathered data using two FGDs, one FGD conducted to obtain the perspective of Puskesmas head and two staff.

The second FGD was conducted in Cilandak sub-district Puskesmas to identify the obstacles faced by both health cadres and Puskesmas staff in carrying out child nutritional health promotion programs and identify suitable promotion programs. We further divided FGD for health cadres into two groups, while FGD for Puskesmas staff was conducted in one group. Each FGD was led by one principal investigator.

A questionnaire-based survey was developed to be completed by health cadres and Puskesmas staff. The questionnaire was used to determine the baseline knowledge, attitude, and practice (KAP) on children's growth and development and their screening at Posyandu. The questionnaire consisted of 22 knowledge-based multiple-choice questions, 18 4-point Likert scale questions to assess attitude, and three yes or no questions to assess practice (Supplementary material 1).

PIs had a discussion with PrimaKu^®^ application development team member and two pediatricians from the scientific division and public relation division of the Indonesian Pediatric Association as application content creators. The discussion was aimed at identifying education material that can be provided through the application to cater to the needs of health cadres in performing their tasks in health promotion and children's nutritional status surveillance. Following this meeting, the team made the final decision on the type of health promotion media to assist the health cadres.

### Phase 2: Video development

A literature review was conducted on Posyandu activities, nutritional requirements during pregnancy and for children aged 1 to 2 years old by searching through search engines such as Google and journal databases such as Medline with keywords including but not limited to the following: “Posyandu”, “nutrition”, “golden period”, “children”, “breastmilk”, “breastfeeding”, “food preparation”. Information from the literature review was used to prepare video content. The duration of each video was around 1 to 2 min. Live-action video footage of real people or actors was taken by cameras. Video editing of footage was done with Final Cut Pro X Version 10.4.6. Video content was reviewed by PIs, a general practitioner from Cilandak sub-district Puskesmas, and pediatricians from the Indonesian Pediatrics Association to ensure the relevance and accuracy of the language and materials within the video.

### Phase 3: Health education using video

All five videos were played in the health cadres training session held at Puskesmas. Health cadres were asked to complete a pre-test before and a post-test after viewing all videos. Pre-test and post-test questions were identical and consisted of 10 multiple-choice and 12 True or False questions, constructed to assess knowledge on nutritional intake for children and Posyandu activities mentioned in the videos. The efficacy of videos as health promotion media was evaluated by comparing scores of pre-test and post-test.

### Data analysis

Questionnaire data were analyzed by converting each response into a score. For knowledge-based questions, participants' answers were categorized as correct or incorrect responses. The incorrect response was scored as 0 and the correct response was scored as 1. For attitude-based questions, answers were graded depending on the statement. For a positive statement, responses were given scores from 5 to 1, which correspond to strongly agree to strongly disagree. While for a negative statement, responses were given scores from 5 to 1 which correspond to strongly disagree to strongly agree. The total score for each participant's attitude was categorized into bad, neutral, or good based on the following scale: “bad” for a total score of < 36 (< 50% of possible maximum score), “neutral” for a score of 36–54 (50–75% of possible maximum score), and “good” for score >54 (>75% of possible maximum score). In behavioral questions, correct behavior was scored as 1 while incorrect behavior was scored as 0. In the pre-test and post-test for video evaluation, the correct response was given a score of 1, and an incorrect response score of 0.

Shapiro-Wilk data normality test with *p* > 0.05 was performed to meet the parametric test requirement. Mean ± standard deviation was used to explain normally distributed numerical data. A comparison of pre-test and post-test participants' total scores was performed with the parametric paired student's *T*-test using a level of statistical significance of < 0.05. All analyses were done with IBM SPSS Statistics for Macintosh, Version 24.0.

## Results

### FGD participants' profile

Three participants were involved in the first FGD, of which two were the Puskesmas staff and one was the Head of Puskesmas. All of them were female with university-level education. The staff's age was between 20–35 years old while the Head of Puskesmas age was older than 35 years old.

Thirty-five participants were involved in the second FGD and filled out the questionnaire, all of whom were female (Table 1). Most of the participants were healthcare cadres (85.7%), followed by Puskesmas staff (14.3%). The participants' age ranged from under 20 years old (3%), between 20–35 years old (11%), and older than 35 years old (86%). Participants' education levels ranged from university (23%), high school (49%), middle school (20%), and elementary school (8%). In total of 77% were employed while the rest were unemployed.

**Table 1:** Demographic profile of FGD participants (*N* = 35).

**Characteristic**	**N (%)**
**Participants' role**
Healthcare cadres	30 (85.7 %)
Puskesmas staff	5 (14.3 %)
**Age**
< 20 years old	1 (2.9 %)
20–35 years old	4 (11.4 %)
> 35 years old	30 (85.7 %)
**Highest education**
University	8 (22.8 %)
High School	17 (48.6 %)
Middle School	7 (20 %)
Elementary School	3 (8.6 %)
**Employment**
Employed	27 (77.1 %)
Unemployed	8 (22.9 %)

### Phase I: Need assessment

From the first FGD, the Puskesmas staff remarked that stunting was one of the major problems they faced in the area. There were at least five out of 4,693 (0.1%) children under 5 years old who had been diagnosed with stunting. They explained issues faced by one of their Posyandu. First, there was a lack of proper educational material that can be used by the Puskesmas. Second, the conventional way of education merely by using static images and texts was outdated. From this data, there was room for improvements to make proper and effective educational material and media.

For the second FGD session, we divided the FGD into a Puskesmas staff group and a health cadres group. FGD was carried out simultaneously and the independent results from these discussions were combined and used for phase 2 in the education content preparation. The FGD with Puskesmas staff concluded that data recording and health cadres' skills and knowledge need improvement. Previously, data recording in Cilandak Sub-district Puskesmas was conducted using e-PPGBM^®^, an application to record nutritional status provided by the Indonesian Health Ministry. However, in practice, cadres recorded the nutritional status on paper, which was then inputted by the Puskesmas staff into e-PPGBM. The double entry of nutrition data into the application and paper-based database was inconvenient and time-consuming for the cadres. Another issue was the quality of data collected by the health cadres is still not reassuring due to a lack of skills and knowledge. Although they had received training, they lacked in managing children with developmental issues and inappropriate feeding practices. Therefore, further education for health cadres and more effective education methods for parents were needed. The ideal health educational media had to be accessible, accurate, and not cadre-dependent, which led us to choose educational video as the most suitable solution.

FGD conducted with health cadres discussed the following topics: challenges for cadres, process of data recording, and opinion regarding application development. Health cadres often encountered challenges such as low attendance of parents to Posyandu due to low enthusiasm and some parents often felt offended after being educated by health cadres. Regarding data recording using e-PPGBM^®^, health cadres also faced the same problem as the Puskesmas staff. Therefore, the majority of health cadres no longer use e-PPGBM for data recording. They need an application that can synchronize to the main database, yet they feel the paper-based recording is still imperative. They also requested health educational material from the application, including methods to calculate corrected age for preterm babies, determine stage development based on corrected age, and stimulate children's development.

**Figure 2:**
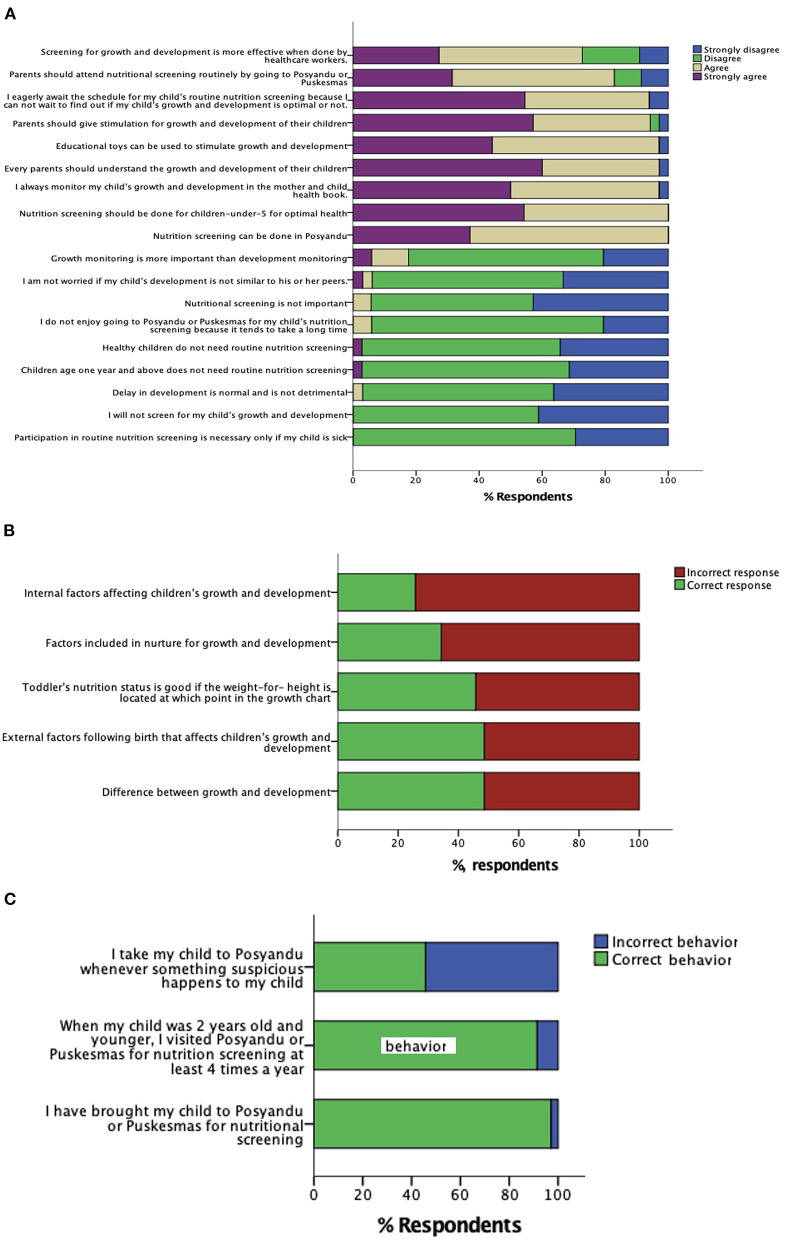
Participants' responses for baseline **(A)** attitude, **(B)** knowledge*, and **(C)** behavior (*n* = 35) on monitoring children's growth and development and utilization of Posyandu as growth and development monitoring facilities. *Only 5 questions with the highest proportion of incorrect responses were represented in the graph.

The baseline attitude of participants was generally good. The majority of participants 68.6% (24/35) had good attitudes towards children's growth and development, 31.4% (11/35) had neutral attitudes, while none of the participants had bad attitudes. Participants' responses to the 18-item questionnaire to evaluate their attitude is shown in Figure 2A. The questions were initially developed for parents, but in this study, the questionnaires were filled by health cadres who acted as a proxy for parents as the final target of health education material produced in this study. Items that yielded the highest proportion of participants' incorrect attitudes were statements about growth and development monitoring. Many participants disagreed or strongly disagreed (31.4%, 11/35) that nutritional screening is more effective when done by healthcare workers. Many also strongly disagreed or disagreed (17.1%, 6/35) to attend the nutritional screening at Posyandu routinely. There were 11.4% (4/35) participants who were not eager to attend Posyandu and find out about their child's growth and development. There were 20% (7/35) of participants who strongly agreed or agreed with the statement that “growth monitoring was more important than development monitoring.”

From the 22 multiple-choice questions to determine participants' baseline knowledge of children's growth and development, the mean total score was 18.1 ± 2.63 out of a full mark of 22. The minimal score was 12 and the maximal score was 22. The top five questions with the highest proportion of incorrect responses from participants were shown in Figure 2B. These questions covered topics of factors affecting children's growth and development, interpretation of growth charts, and differences between growth and development.

For baseline behavior of participants (Figure 2C), the majority of the participants displayed incorrect behavior only on one out of three items. In total of 54.3% (19/35) participants did not go to Posyandu whenever they suspected something abnormal in their children's health.

### Phase II: Video development

The video development process was initiated by analyzing the need assessment results. Based on the result of participants' attitudes, it was found that items that yielded the highest proportion of participants' incorrect attitudes were statements about growth and development monitoring. In addition, the KAP survey on the attitude component revealed that 17.1% did not agree that Posyandu needs to be attended regularly. Related to these results, the researchers decided to develop a video explaining the activities in Posyandu to encourage routine attendance.

From the second FGD, it was found that the health cadres had limited knowledge of nutritional intake and skills in managing inappropriate feeding practices. Therefore, the team decided to develop educational videos about nutritional intake.

Although PrimaKu^®^ already had educational material about nutrition to support children's growth and development, mostly it was text-based. Thus, there was a need to visualize the material through educational videos based on the data collection in the FGD session.

In total, there were five videos produced: a video about regular activities in Posyandu; and four videos elaborating on children's nutritional intake recommendations in four different age groups, which are birth to 6 months (breastfeeding), 6–9 months, 9–12 months, and 1–2 years old. Each video lasted 1 to 2 min. One of the videos was about breastfeeding recommendations for 0–6 months children, showing the proper breastfeeding process, breast milk storage, and breast milk preparation. Whereas three other videos for different age groups demonstrated the suitable complementary food-making process, types, and amount of macronutrients and micronutrients that were recommended to the age group, and introduced food varieties. These videos with English subtitles are available on YouTube with links provided in the Supplementary material 2.

Firstly, we chose several food menus that consisted of balanced macronutrients and micronutrients. Then, we prepared the food ingredients. Secondly, the ingredients were properly cooked with healthy and hygienic procedures, such as boiling and steaming. Thirdly, the cooked ingredients were processed by cutting, chopping, and mashing. This was done to highlight the significance of cooking methods in producing proper texture and consistency that are appropriate for children's development stage in food mastication and digestion. In addition, we emphasized the frequency of feeding. Through these videos, we expect the cadres and mothers to easily understand and implement these examples.

### Phase III: Education using video

The pre-tests were completed by 20 health cadres. The mean score was 18.08 ± 0.423, with a minimum score of 13/22 and a maximum score of 21/22 (Table 2). Five questions with the lowest correct responses were about Posyandu station for counseling and education (12%), fluid requirement for children (24%), correct methods of breastfeeding (28%), and lastly, direct and indirect breastfeeding methods (both at 68%). All participants responded correctly to three questions: number of posyandu stations, growth disturbance reporting system, and variation of complementary foods.

**Table 2:** Pre-test and Post-test results (*N* = 20).

**Questions**	**Correct response (%)**		
	**Pre-test**	**Post-test**	**Delta**
Posyandu service target	92	92	0
Posyandu schedule	92	96	+4
Posyandu executor	96	96	0
Posyandu activities	88	92	+4
Number of Posyandu stations	100	100	0
Posyandu station for toddler	84	84	0
Posyandu station for counseling and education	12	16	+4
Recording of Posyandu assessment	96	96	−4
Growth disturbance reporting system	100	96	+4
Exclusive breastfeeding target	96	100	+48
Correct methods of exclusive breastfeeding	28	76	+4
Breast side to start breastfeeding	68	72	+4
Indirect breastfeeding method	68	92	+24
Breastfeeding frequency	84	92	+8
Breastfeeding timing	96	92	0
Breastmilk storage	88	92	+4
Timing for initiating complementary foods (*MPASI)*	92	92	0
Complementary foods (MPASI) method	96	100	+4
Complementary foods (MPASI) for children aged 12–24 months old	76	88	+12
Variation of complementary foods (*MPASI)*	100	100	0
Timing for initiating table food	92	100	+8
Fluid requirements for children	64	96	+32

After watching the videos, the same 20 health cadres completed the post-test (Table 2). The mean score improved to 19.60 ± 0.311, with a minimum score of 16/22 and a maximum score of 22/22. The correct responses to the question regarding Posyandu station for counseling and education remained the lowest (16%). Among the five questions with the lowest correct responses in the pre-test, four of them were improved: correct methods in giving exclusive breastfeeding, breast side to start breastfeeding, fluid requirement, and indirect breastfeeding method. However, two of the former still had the lowest proportion of correct responses in the post-test with 76 and 72%, respectively. All participants responded correctly to five questions which were the number of Posyandu stations, variation of complementary foods, the target of exclusive breastfeeding, methods of complementary foods, and timing for initiating table food. Shapiro-Wilk test showed that the data was in a normal distribution (*p*-value = 0.18). *T*-test results showed that there was a significant improvement from pre-test total scores to post-test total scores (effect size = 0.88, *p*-value = 0.00).

## Discussion

Indonesia is lagging behind its neighboring countries in the race to reduce stunting. The health cadres require further assistance in performing their tasks as the frontliners for health promotion and children's nutrition surveillance. With the growing use of the internet and mobile phones in Indonesia, an app-based tool is a potential solution to this problem (8). This study aimed to identify features on a mobile application and develop educational materials that can be available on an existing mobile application, to empower cadres in their health promotion activities.

Empowerment is a process of gaining understanding and control of personal, social, economic, and political status to take action that can improve one's health. This definition applies in personal and community settings. Empowerment is the core principle of health promotion endorsed by WHO. Empowerment includes a strategy that advocates participation, builds awareness, and form problem-solving skills and targets groups that are socially disadvantaged (10). The target of the empowerment program in this study, are cadres who primarily have low level of education, with 72% of the cadres education level below university.

The study was conducted in three phases; the researchers looked at the results from the previous phase as a basis for activities in the next phase. The final target of the health promotion in this study is the community parents who attend Posyandu. However, the empowerment is targeted at health cadres as community leader that will further use their skills and knowledge to educate community parents. The need assessment was performed on the cadres because cadres are women who are recruited from the same community that they serve. Their knowledge, attitude and behavior would reflect that of the community parents. Based on the FGD and KAP assessment, the challenges faced by cadres could be categorized as (1) difficulties in data recording and maintenance using the new electronic database; (2) limitations of skills and knowledge in assessing growth and development; (3) limitation of knowledge on appropriate feeding practice and development stimulation; (4) resisting attitude to health promotion and attendance to routine nutrition screening; and (5) misperception that growth is more important than development.

This study attempted to conduct training of trainers in the absence of on-site trainers but *via* mobile applications that can be readily available for cadres and parents. Since data recording and maintenance as well as growth and development assessment are skills that require hands-on training, this study focused to train cadres on topics that can be trained without direct supervision. We focused on improving cadres knowledge on areas where they are lacking and on habits that are not health-promoting *via* educational media that can be readily available on a mobile application.

The current Posyandu is only held once a month thus limiting parents' access to information regarding children's growth and development. The cadre needs a health promotion tool that is easily accessible, accurate, and readily available for the parents. Video utilization as educational tools is supported by Schmid et al. study which concluded that technology use in education could enhance the learning process (11). Another study by Moore et al. found that video had comparable effectiveness in the education process as a live demonstration (12). In addition, Rackaway et al. found that video could significantly improve learning outcomes (13). The researchers decided that video available through an application will be a suitable solution for the cadre's needs. The video can be available for free on mobile phones and will be easily accessible by parents. It will also convey accurate information by avoiding the possibility of passing on false information by cadres who were not knowledgeable.

In 2020, the Central Agency on Statistics reported that 78.18% of households in Indonesia had already used the Internet daily. The high penetration rate of the Internet in Indonesia supports the development of internet-based education materials. Additionally, Setyastuti et al. showed that 55.4% of young mothers in Indonesia preferred internet-based educational material, further supporting video utilization as an educational tool. Videos produced in this study were made to help the cadre in providing intervention for children with inappropriate feeding practices. Four videos were made for four different age groups: 0–6 months, 6–9 months, 9–12 months, and 1 to 2 years old. These age groups were selected because studies had shown nutrition during this period was an important determinant of medical and socioeconomic status in adulthood (4). Optimal nutrition during this period is also crucial to prevent stunting (4). One video was made to encourage visits to Posyandu to correct the resisting attitude of parents to health promotion and routine attendance.

The parents who rely on Posyandu for their children's health are mostly of low socioeconomic and educational backgrounds. Thus, it is particularly a challenging task to promote health to this population. In addition, Indonesia, being a developing country, may lack accessible facilities and well-trained health workers to cater to the needs of the low-income population (14). Studies have shown that videos are effective as low-cost interventional tools. Videos on health promotion that target developing nations' populations or low-income populations had been shown to significantly improve the knowledge of the target audience (15, 16). The cadre also demonstrated a significant improvement in knowledge after watching the educational videos (*p* = 0.00).

The final target of the education material is the parents; however, the video was made to help cadres in delivering accurate information to the parents. The educational videos do not replace nor make the health cadres lose their role to explain and guide parents on nutritional aspects for children. Health cadres are expected to provide examples and be actively involved as community nutritional consultants using information that they have gained from the videos and all the training they've done. Therefore, the importance of making this video is for complementing the essential role of cadres as health promoters. Once the information from the video is delivered directly by the health cadres, parents may ask cadres for further clarifications on information that they did not understand yet or any other information that was not available in the videos. Parents may also use the video to review the acquired information at home, further minimizing errors that could happen in implementing the provided information in their own home.

One video was created to encourage Posyandu's visit due to findings from FGD where the cadre felt parents lack the enthusiasm to attend Posyandu. In addition, the survey on baseline attitudes of cadres revealed that 17% disagreed or strongly disagreed to attend Posyandu routinely for nutrition screening. The survey also showed a majority (54.3%) of cadres did not go to Posyandu when they suspect something is abnormal with their children's health. This attitude and behavior of the cadre can be used to extrapolate that of mothers who attend Posyandu because most cadres were mothers in the community themself. The reasoning behind this extrapolation is that cadres are community health workers which WHO defines as “community health aides selected, trained and working in the communities from which they come”. As community members, their socio-economic background is similar to the women or parents that are the final target audience of the educational videos, thus we could expect similar attitudes and behavior from the parents (17). Thus, these findings suggest there is a need to improve the understanding of cadre and parents on the importance of attending Posyandu routinely.

In the current Posyandu practice, generally, only growth monitoring is performed. There is no station in the current Posyandu practice dedicated to monitor development, hence children's development is barely monitored. This is reflected in our study as 20% of cadres feel that growth monitoring is more important than development monitoring. This lack of children development monitoring is an issue that is realized by the cadre. In FGD, the cadre requested tools that can help them monitor children's development and provide information on the age-appropriate stimulation for the different children's developmental stages.

With the recent advent of mobile-based applications, the government has also provided the cadre with an application-based tool called e-PPGBM^®^. The aforementioned application was able to aid health cadres in growth data recording. However, the results from our study showed that these cadres feel e-PPGBM lacks several features, especially the health education tools.

The current Indonesian Pediatric Association's smartphone application for child growth monitoring and education contained educational materials such as nutritional recommendations for growth and stimulation cues for motoric, language, and social development. However, these materials were still in text form with an addition of a few pictures provided to visualize stimulation cues for each aspect. Through this study, we have produced five videos to be added to the Primaku^®^ application to provide a more effective visual education on nutrition and Posyandu visits. Thus, for our future work, we plan to produce videos that demonstrate development stimulation cues. These videos can be added to Primaku^®^ app for the health cadres to aid them in evaluating and monitoring children's development.

The strength of this study lies in the analysis of both quantitative and qualitative data to determine the needs of cadres and to provide the cadres with tools that can ease them in their function as community health promoters. To the authors' knowledge, this is the first published study in Indonesia that produces education tools to aid cadres through a systematic quantitative and qualitative analysis.

Kirkpatrick (18) developed an evaluation framework to comprehensively assess the efficacy of the training program. This evaluation framework consists of evaluation of (1) reaction (2) learning (3) behavior and (4) results. The first level is where reactions from trainees are evaluated, this is necessary to gain feedback on instructors or materials provided. The second is the extent of learning or knowledge gained that can be assessed with pre-test and post-test evaluation. The third level evaluates the application of that knowledge into a habit, while the fourth level evaluates results, or how the training impacts the main goal of the program. The main goal of training in this study is to reduce the stunting rate in the sub-district. In this only learning is evaluated. According to the model of behavior change by Allport, behavior change starts with the acquisition of knowledge, the generation of attitude, and later will lead to the formation of practice that is in agreement with the attitude. Based on this model there is a high positive correlation between knowledge and attitude or between attitude and behavior (19). This study only evaluated knowledge because it is the first component that could immediately be evaluated in this study based on this theory as the post-test was conducted immediately after the video showing. A further evaluation of attitude and practice may be done if the evaluation was done on a different day or time from the video viewing. For an action to become a habit, the action needs to attain automaticity. A study by Lally et al. that measures the duration for an action to be performed automatically found that on average it took around 66 days or around 10 weeks to become a habit (20). Therefore in future studies it may be necessary to follow up on whether the knowledge shared through the videos leads to a change in behavior in a 10-week duration follow-up.

To improve the efficacy of the training method, Kirkpatrick explained 4 levels of evaluation that should be performed. This study has only used the second level that is the evaluation of learning. It may be necessary to evaluate the first level that is the reaction because positive reaction motivates learning, and at later times evaluate behavior change, ideally in 10 weeks where habits tend to form. At later time stunting rate as the main goal of the program should be evaluated so that the whole aspect of the training program can be assessed. This study aimed to create educational material that helps cadres in educating parents on child nutritional management. However, the efficacy of the education material was only assessed on cadres as the deliverer information, future studies assessing video efficacy on parents as the final target of health education are necessary. Another limitation of this study is this study did not directly address the issues of lack of understanding on the importance of development as well as skills in monitoring child's growth and development. Future work to address this problem is needed to provide a holistic solution to the stunting problem in Indonesian district communities and eventually nationally.

## Conclusion

Stunting is a major health issue in Indonesia. From FGDs with Puskesmas staff and health cadres, there was a need for effective educational material on nutrition and nutritional status data recording platform. Through this study, videos on the importance of Posyandu routine visits and children under 2 years old nutritional intake recommendations were made. There was a significant improvement in the knowledge of cadres following educational video viewing. This study has shown that smartphone application-based educational videos about children's nutrition are effective as health promotion media for Puskesmas staff and reliable health information on demand for parents.

## Data availability statement

The original contributions presented in the study are included in the article/Supplementary material, further inquiries can be directed to the corresponding author/s.

## Ethics statement

Ethical review and approval was not required for the study on human participants in accordance with the local legislation and institutional requirements. The patients/participants provided their written informed consent to participate in this study.

## Author contributions

DF and AK: conceptualization, funding acquisition, methodology, project administration, supervision, and visualization. FR: conceptualization, data curation, formal analysis, visualization, writing-original draft, and writing-review and editing. AR: conceptualization, formal analysis, visualization, writing-original draft, and writing-review and editing. NI: data curation, formal analysis, visualization, writing-original draft, and writing-review and editing. EA, NU, and AW: formal analysis, project administration, visualization, writing-original draft, and writing-review and editing. RA: project administration and supervision. All authors contributed to the article and approved the submitted version.

## Funding

This study was funded by a community service grant given by the Directorate of Research and Community Engagement, Universitas Indonesia.

## Conflict of interest

The authors declare that the research was conducted in the absence of any commercial or financial relationships that could be construed as a potential conflict of interest.

## Publisher's note

All claims expressed in this article are solely those of the authors and do not necessarily represent those of their affiliated organizations, or those of the publisher, the editors and the reviewers. Any product that may be evaluated in this article, or claim that may be made by its manufacturer, is not guaranteed or endorsed by the publisher.
