# Supplementary material for: Health cadres empowerment program through smartphone application-based educational videos to promote child growth and development
Source: Front Public Health. 2022 Oct 13;10:887288. doi: 10.3389/fpubh.2022.887288 (PMC9611201; doi:10.3389/fpubh.2022.887288)
Supplement: Supplementary file 1 [file Data_Sheet_1.DOCX]

**Supplementary File 1**

**Translated Questionnaire**

Mother’s Knowledge, Attitude, and Behavior on Children’s Growth and Development in Cilandak Sub-district Puskemas

**A. Personal Information**

1. Name (Initial):

2. Address:

3. Sub-district:

4. Role

❏ Puskesmas staff, division …...

❏ Healthcare cadres

5. Age:

❏ < 20 years old

❏ 20 – 35 years old

❏ > 35 years old

6. Highest education:

❏ No formal education

❏ Elementary school

❏ Middle school

❏ High school

❏ University

7. Employment :

❏ Employed, job: ……

❏ Unemployed

8. Child’s age (skip this question if you don’t have a child)

❏ 0 – 6 months old

❏ 6 – 12 months old

❏ 12 – 23 months old

❏ 24 – 36 months old

❏ 37 – 48 months old

❏ 49 – 60 months old

9. Source of information for growth and development monitoring

❏ Personal experience

❏ Other people

❏ Culture (from generation to generation)

❏ Media (television, radio, newspaper, Internet)

❏ Formal education (school, university)

❏ Belief system (religion)

❏ Personal perception

**B. Knowledge**

1. The term growth and development is a...

a. Same event but have a different meaning

b. Different events but have the same meaning

c. Different but related events

2. What is the definition of growth?

a. The increase in the size of cells and tissues, physical size which can be measured in units of length and weight

b. Physiological and functional maturation that occur in an orderly pattern

c. Increased physical size and more complex body functions

3. What is the definition of development?

a. The increase in the size of cells and tissues, physical size which can be measured in units of length and weight

b. Physiological and functional maturation that occur in an orderly pattern

c. Increased physical size and more complex body functions

4. Internal factors that can affect growth and development include...

a. Age, family, and gender

b. Nutrition, psychology, and infection

c. Environment, stimulation, and socioeconomic

5. Postpartum external factors that can affect growth and development include...

a. Nutrition, psychology, and infection

b. Environment, stimulation, and socioeconomic

c. Age, family, and gender

6. Nurturing aspects of growth and development include...

a. Stimulating child’s potentials (*asah*) and fulfilling the needs of the child (*asuh*)

b. Providing affection (*asih*) and fulfilling the needs of the child (*asuh*)

c. Providing affection (*asih*), stimulating child’s potentials (*asah*) and fulfilling the needs of the child (*asuh*)

7. Which of the following is a part of *asah* aspect?

a. Nutrition, clothing, and home

b. Giving sensory, motor and cognitive stimulation

c. Support, love, and security

8. Aspects of developmental tasks include...

a. Cognitive, motor, and language

b. Personal, social, and emotional

c. All of the above are correct

9. Gross motor aspects include...

a. Walking, jumping and running

b. Writing, drawing and pointing

c. Communicating and singing

10. Fine motor aspects include...

a. Socializing

b. Writing, drawing and pointing

c. Walking, jumping, and running

11. Cognitive aspects include...

a. Thinking, talent, and intelligence

b. Walking, jumping, and running

c. Interacting with others

12. The oral stage occurs at the age of...

a. 8 months old

b. 9 months old

c. 1-1.5 years old

13. Growth and development for children aged 12-18 months include these stages, except for:

a. Standing without support

b. Inserting a cube into a box

c. Taking off their clothes

14. Stages of growth and development at the age of 24 months include:

a. Saying 3-6 words that have meaning

b. Stacking 2 cubes

c. Showing jealousy

15. Stages of children aged 36 months include...

a. Walking up the stairs alone

b. Eating on their own without spilling

c. All of the above are correct

16. What is stimulation?

a. Activities to stimulate basic abilities so that children grow and develop optimally

b. Invite to play

c. Monitor growth and development

17. Examples of stimulation of speech and language skills in children aged 24-36 months include...

a. Reading them a storybook

b. Singing together

c. All of the above are true

18. Developmental disorders include...

a. Impaired physical growth, impaired motor development

b. Impaired language development, emotional and behavioral disorders

c. All of the above are correct

19. Examples of physical growth impairments include:

a. Obesity, microcephaly, microcephaly, short stature

b. Autism, loss of appetite

c. Down syndrome, cerebral palsy

20. Examples of emotional and behavioral disorders include...

a. Autism

b. Lack of social interaction

c. Macrocephaly

21. When should the baby be breastfed for the first time?

a. One day after the baby is born

b. When the baby starts to cry

c. Immediately after the baby is born

22. Complementary feeding food should be given gradually, for example...

a. Started from thin puree to thick puree

b. Started from thick puree

c. Started from thick puree to ground rice

23. If the results of the height measurement on the *Kartu Menuju Sehat* (KSM or Towards Healthy Card) are below the red line, then what we should do is to...

a. Provide additional food that can support the growth of toddlers

b. Continue eating pattern as usual

c. Reduce the food portion

24. The child's weight growth is considered to increase in the KMS if:

a. The weight line is increasing

b. The weight line is decreasing

c. The weight line is flat

25. The nutritional status of children under-five years old is considered good if the point that indicates the weight is …… the height growth line on the growth graph.

a. Above

b. Exactly at

c. Under

**C. Attitude**

| **No** | **Statement** | **Strongly Agree** | **Agree** | **Disagree** | **Strongly Disagree** |
| --- | --- | --- | --- | --- | --- |
| 1 | Children aged one year and above do not need routine nutrition screening |  |  |  |  |
| 2 | Nutrition screening can be done in Posyandu |  |  |  |  |
| 3 | Nutrition screening should be done for children under 5 for optimal health |  |  |  |  |
| 4 | Nutritional screening is not important |  |  |  |  |
| 5 | Parents should attend nutritional screening routinely by going to Posyandu or Puskesmas |  |  |  |  |
| 6 | Healthy children do not need routine nutrition screening |  |  |  |  |
| 7 | Participation in routine nutrition screening is necessary only if my child is sick |  |  |  |  |
| 8 | Parents should give stimulation for the growth and development of their children |  |  |  |  |
| 9 | Educational toys can be used to stimulate growth and development |  |  |  |  |
| 10 | Every parent should understand the growth and development of their children |  |  |  |  |

| 11 | Delay in development is normal and is not detrimental |  |  |  |  |
| --- | --- | --- | --- | --- | --- |
| 12 | I am not worried if my child's development is not similar to his or her peers |  |  |  |  |
| 13 | I will not screen for my child's growth and development |  |  |  |  |
| 14 | I eagerly await the schedule for my child’s routine nutrition screening because I can not wait to find out if my child’s growth is optimal or not |  |  |  |  |
| 15 | I do not enjoy going to Posyandu or Puskesmas for my child's nutrition screening because it tends to take a long time |  |  |  |  |
| 16 | I always monitor my child's growth and development in the mother and child  health book. |  |  |  |  |
| 17 | Screening for growth and development is more effective when done by healthcare workers. |  |  |  |  |
| 18 | Growth monitoring is more important than development monitoring |  |  |  |  |

**D. Behaviour**

| **No** | **Statement** | **Yes** | **No** |
| --- | --- | --- | --- |
| 1 | I have brought my child to Posyandu or Puskesmas for nutritional screening |  |  |
| 2 | When my child was 2 years old and younger, I visited Posyandu or Puskesmas for nutrition screening at least 4 times a year |  |  |
| 3 | I take my child to Posyandu whenever something suspicious happens to my child |  |  |
