# Supplementary material for: Health cadres empowerment program through smartphone application-based educational videos to promote child growth and development
Source: Front Public Health. 2022 Oct 13;10:887288. doi: 10.3389/fpubh.2022.887288 (PMC9611201; doi:10.3389/fpubh.2022.887288)
Supplement: Supplementary file 2 [file Data_Sheet_2.docx]

**Supplementary File 2**

1. Let’s go to Integrated Health Post (Posyandu) : <https://www.youtube.com/watch?v=0FXdf1HBxcc>
2. Breastmilk :

<https://www.youtube.com/watch?v=RrIh95ikRkI>

1. Complementary foods for infant age 6 to 9 months old:

<https://www.youtube.com/watch?v=Yyu3BDMde-w>

1. Complementary foods for infant age 9 to 12 months old:

<https://www.youtube.com/watch?v=eGEquG4xAj8>

1. Complementary foods for child age 12 months old to 2 years old

<https://youtu.be/Tkh0TRe-0b8>

For subtitle in English, on video select **CC (closed captions)** , and select **Settings** 🡪 **Subtitles** 🡪 **English**
